# Quantities and units for metabolic processes as a function of time (Recommendations 1992)

**DOI:** 10.1155/S1463924692000415

**Published:** 1992

**Authors:** G. Férard

**Affiliations:** Laboratoire de Biochimie appliquée Université Louis Pasteur, F-67401 Strasbourg France

## Abstract

In order to standardize time-related reports in clinical chemistry, basic concepts like system, component and kind-of-quantity are recalled. Changes of a quantity, and of chemical and physical processes involved in these changes are refined. Starting from the general format recommended by IUPAC and IFCC, including system, component and kind-of-quantity, recommendations are formulated. Specifications, especially time-related information, may be added in parentheses after each part of the format. Interrelated specifications are given after the kind-of-quantity. This paper provides examples of various specifications which show that all these elements are necessary for reporting time-related data.


Journal of Automatic Chemistry, Vol. 14, No. 6 (November–December 1992), pp. 231–234

### Quantities and units for metabolic processes as a function of time (Recommendations 1992)

**G. Férard, on behalf of the International Union of Pure and Applied Chemistry, Clinical Chemistry Division, Committee on Quantities and units in Clinical Chemistry and the International Federation of Clinical Chemistry, Scientific Division Committee on Quantities and Units**

*Laboratoire de Biochimie appliquée, Université Louis Pasteur, F-67401 Strasbourg, France*

*In order to standardize time-related reports in clinical chemistry, basic concepts like system, component and kind-of-quantity are recalled. Changes of a quantity, and of chemical and physical processes involved in these changes are refined. Starting from the general format recommended by IUPAC and IFCC, including system, component and kind-of-quantity, recommendations are formulated. Specifications, especially time-related information, may be added in parentheses after each part of the format. Interrelated specifications are given after the kind-of-quantity. This paper provides examples of various specifications which show that all these elements are necessary for reporting time-related data.*

### 1. Introduction

Time-related measurements are performed in a variety of clinical chemistry investigations, for example endocrine stimulation or suppression tests, in nutritional or therapeutic drug-loading test, in monitoring physiological and pathological processes and the excretion or secretion of components, and in measuring catalytic activities of an enzyme in a system. The basis of the understanding of the test results lies in biochemical, physiological, pharmacological and pathological studies.

Because of the differences between tests and their applications, results have until now been presented in a variety of ways in clinical chemistry.

This document aims to:

(1) Analyse the elements of the specifications for time-related measurements in the clinical laboratory.

(2) Recommend a format that will unify, simplify, clarify, and, hence, improve the reporting and understanding of time-related quantities measured in the clinical laboratory.

### 2. Basic concepts, their terms and definitions

*2.1.* The *concepts* defining a quantity comprise system, component, kind-of-quantity, numerical value and unit [1].

A *quantity, Q,* is a measurable, real property, physical or chemical, of a specified system. Example: substance concentration of triglycerides in the blood plasma of a named patient at a given date.

A *system* is a term that may be applied to any arbitrarily chosen, but stated part of the universe irrespective of form or size. Examples: a specified patient, a tube of plasma.

A *component* is a stated part of the system.

A *kind-of-quantity* is the abstract concept of the property common to a number of real phenomena (quantities); an example is substance concentration.

*Numerical value* is the number that gives the magnitude of the measured quantity when multiplied by the unit.

*Unit* is a chosen reference quantity which may be used for comparison of quantities of the same kind. An example is mole (symbol: mol).

It is customary to consider that the quantity, $Q$, is expressed by the product of a numerical value, $\{Q\}$, and an appropriate unit, $[Q]$, also called the value of the quantity:

$$Q = \{Q\} \cdot [Q].$$

When speaking about the differential of a quantity, $dQ$, it is the differential of its value that is being described.

*2.2. Change* of a quantity is the increment of the value of $Q$ with time. The change may be expressed either infinitesimally at time by the differential $dQ$ or $dQ(t)$, or in practice it may be expressed by a finite increment over the time interval $(t_1; t_2)$, that is $Q(t_2) - Q(t_1)$ which may be written $\Delta Q$ or $\Delta Q(t_1;t_2)$:

$$\Delta Q = Q(t_1;t_2) = Q(t_2) - Q(t_1).$$

Examples are mass change, $\Delta m$; amount-of-substance change (short form: substance change), $\Delta n$; volume change, $\Delta V$; substance concentration change, $\Delta c$.

Note: *Net change* of a quantity in a system is the algebraic sum of the changes of the quantity affected by different processes:

$$dQ \text{ net} = \sum_{i=1}^{n} dQi$$

or in practice it may be expressed by a finite time interval $(t_1;t_2)$:

$$\Delta Q(t_1;t_2)\text{net} = \sum_{i=1}^{n} \Delta Qi(t_1;t_2).$$

*2.3. Fractional change* of a quantity may be expressed infinitesimally at time $t$ by the differential $dQ(t)/Q(t)$. For

© IFCC 1992

a finite time interval the quotient is:

$$\Delta Q(t_1;t_2)/Q(t_1) = [Q(t_2) - Q(t_1)]/Q(t_1).$$

Note: The quantities $Q(t_1)$ and $Q(t_2)$ are of the same kind and have the same type of component.

Note 2: Fractional change has dimension one.

Examples: Mass fractional change, $\mathrm{d}m(t)/m(t)$; amount-of-substance fractional change, $\mathrm{d}n(t)/n(t)$ (short form: substance fractional change); amount-of-substance fractional change of concentration, $\mathrm{d}c(t)/c(t)$ (short form: substance fractional change of concentration).

*2.4. Change ratio* of a quantity may be expressed infinitesimally at time $t$ by a ratio of differentials $\mathrm{d}Q_1(t)/\mathrm{d}Q_2(t)$, where the kind-of-quantities are the same but for different components in the same system. In practice, the ratio for a finite time interval, is:

$$\Delta Q_1(t_1;t_2)/\Delta Q_2(t_1;t_2).$$

Note: Change ratio has the dimension one.

Examples: mass change ratio, $\mathrm{d}m_1(t)/\mathrm{d}m_2(t)$; amount-of-substance change ratio, $\mathrm{d}n_1(t)/\mathrm{d}n_2(t)$ (short form: substance change ratio); amount-of-substance concentration change ratio, $\mathrm{d}c(t)/\mathrm{d}c_2(t)$ (short form: substance concentration change ratio).

*2.5. Rate of change* of a quantity is defined by the time derivative $\mathrm{d}Q/\mathrm{d}t$ of the value of the quantity. The differential quotient may also be called derivative (or instantaneous) rate of change.

Examples: rate of change of mass, $\mathrm{d}m/\mathrm{d}t$ (short form: mass rate); rate of change of amount-of-substance, $\mathrm{d}n/\mathrm{d}t$ (short form: substance rate); rate of change of amount-of-substance concentration, $\mathrm{d}c/\mathrm{d}t$ (short form: substance concentration rate).

The change in a quantity is often measured as the difference between the values at the two ends of a calendar time interval. Then, the mean rate of change is defined as:

$$[(Q(t_2) - Q(t_1)]/(t_2;t_1) = \Delta Q/\Delta t.$$

Note 1: The modifier 'mean' may be omitted if the time interval is indicated in the quantity name.

Note 2: If the value of a quantity varies linearly with time, then the rate of change remains constant; the mean rate of change, therefore equals the derivative rate of change:

$$\Delta Q/\Delta t = \mathrm{d}Q/\mathrm{d}t.$$

Note 3: For mono-exponential changes, the logarithm of the value of the quantity varies linearly with time – therefore the logarithmic rate of change equals logarithmic derivative rate of change.

$$\Delta \ln Q/\Delta t = \mathrm{d} \ln Q/\mathrm{d}t.$$

*2.6. Rate-of-change ratio* is the quotient of two rates where the quantities are of the same kind in the same system for different components:

$$(\mathrm{d}Q_1/\mathrm{d}t)/(\mathrm{d}Q_2/\mathrm{d}t).$$

For finite time intervals, mean rate of change ratio is:

$$(\Delta Q_1/\Delta t)/(\Delta Q_2/\Delta t) = (\Delta Q_1/\Delta Q_2)\Delta t.$$

Note 1: Rate-of-change ratio has the dimension one.

Note 2: The denominator is often called the reference quantity.

Examples: mass rate ratio $(\mathrm{d}m_1/\mathrm{d}t)/(\mathrm{d}m_2/\mathrm{d}t)$; amount-of-substance rate ratio $(\mathrm{d}n_1/\mathrm{d}t)/(\mathrm{d}n_2/\mathrm{d}t)$ (short form: substance rate ratio); amount-of-substance concentration rate ratio $(\mathrm{d}c_1/\mathrm{d}t)/(\mathrm{d}c_2/\mathrm{d}t)$ (short form: substance concentration rate ratio).

### 3. Processes and related quantities

A *process* is a phenomenon by which change takes place in a system. In physiological systems, a process may be chemical, physical or both.

*3.1. Chemical processes*

*3.1.1. Conversion*
A component may be converted (formed or consumed) by a chemical reaction in a system.

*3.1.2. The extent of reaction,* $\xi$, is the change of amount-of-substance for a formed component, B, divided by the stoichiometric coefficient $\nu_B$:

$$\xi = \Delta n_B/\nu_B.$$

The extent is calculated from the start of the reaction. It is only valid for a time-independent stoichiometry.

*3.1.3. Rate of conversion,* $\dot{\xi}$, is the time derivative of amount-of-substance for a product component B divided by the stoichiometric coefficient $\nu_B$:

$$\dot{\xi} = \mathrm{d}\xi/\mathrm{d}t = (1/\nu_B) \cdot (\mathrm{d}n_B/\mathrm{d}t).$$

*3.1.4. Rate of reaction,* $v$, is the rate of conversion divided by the volume, $V$, of the system in which the process occurs:

$$v = \dot{\xi}/V = (1/\nu_B) \cdot (1/V) \cdot (\mathrm{d}n_B/\mathrm{d}t).$$

This definition is valid for a reaction in which the volume varies with time or for a reaction involving two or more phases [3].

When the volume does not vary with time, the rate of reaction may be expressed in terms of substance concentration:

$$v = (1/\nu_B) \cdot (\mathrm{d}c_B/\mathrm{d}t)$$

where $\xi$ and $v$ are by definition quantities with positive values.

Note: When the changes involved in the quantities defined in sections 3.1.3 and 3.1.4 are only measured at the two ends of a calendar time interval, rates may be called mean rates, i.e. mean rate of reaction, mean rate of conversion.

*3.2. Physical processes*

*3.2.1. Transfer* is the movement of a component either within a system or across its boundary. Transfer may be expressed using different kinds-of-quantities, for example rates of change:

$$\mathrm{d}Q/\mathrm{d}t \text{ or } \Delta Q/\Delta t.$$

Examples: mass rate, $\mathrm{d}m_B/\mathrm{d}t$ or $\Delta m_B/\Delta t$; substance rate, $\mathrm{d}n_B/\mathrm{d}t$ or $\Delta n_B/\Delta t$; volume rate, $\mathrm{d}V_B/\mathrm{d}t$ or $\Delta V_B/\Delta t$.

Transfer of a component may be specified in relation to the system: ***input***, ***intake***, and ***absorption*** are transfer of a component into the system under consideration; *Output*, *excretion* and *secretion* are transfer of a component out of the system under consideration.

*3.2.2. Movement* of a component with respect to a surface or interface may be expressed by:

$$(\mathrm{d}Q/\mathrm{d}t) \cdot (1/A) \text{ or } (\Delta Q/\Delta t) \cdot (1/A)$$

where $A$ is the area of the surface. It is sometimes called flux, but the term flux is used in some disciplines for the kinds-of-quantity defined in section 3.2.1. The concept may be expressed by areic mass rate, areic substance rate, or areic volume rate [4].

*3.3. Combined processes*

The combination of two or more processes (chemical and/or physical) is usual in physiological systems. In that case, a net change may be calculated (see section 2.2).

### 4. Recommendations

*4.1.* The base SI unit for time is the second (symbol s). In the expression of time-related quantities, the second should be used whenever possible, in order to retain the advantages of coherent SI units. Minute (min), hour (h) and day (d) are recognized for use with SI, because of their importance and widespread use [2]. However, in compound units, the use of minutes, hours and days should be limited. It is recommended that the same unit should be used for time throughout the presentation of a set of data in order to simplify the comparison of different quantities.

*4.2.* To improve the understanding of results in clinical chemistry, a general format for reporting results was formulated [1,4]. System–component; kind-of-quantity = numerical value · unit.

Such a presentation of a result is adequate for a comprehensive report, provided the purpose is to assess the patient's status at the moment in time when the sample was obtained.

Specifications, especially time specifications, may be added for the system, the component or for the kind-of-quantity:

System (specifications)–component (specifications); kind-of-quantity (specifications) = numerical value · unit. Interrelated specifications are given after the kind-of-quantity.

*4.3.* In clinical chemistry, each report must contain an indication of the day and time at which the sampling was performed. Further time specifications may be necessary:

*4.3.1.* When a previous event is known to modify the property measured: System–component; kind-of-quantity (time, event) = numerical value · unit.

Examples: Plasma–glucose; substance concentration (60 min after an oral load of glucose = 278 mmol) = 8·5 mmol/l; or

Plasma–glucose; substance concentration change (60 min after an oral load of glucose = 278 mmol) = +3·5 mmol/l.

The same format of presentation should be used for suppression or stimulation tests.

*4.3.2.* When a mean rate is reported, the length or the time limits of the time interval over which the measurements are made need to be stated.

*4.3.2.1.* The length of the time interval should be stated in seconds (min, h, d):

System–component; kind-of-quantity ($\Delta t$) = numerical value · unit.

Example: Patient urine–hydroxyproline; substance rate (3 d) = 150 μmol/d.

*4.3.2.2.* The limits of the time interval should be stated when short-term variations in the measured property as a function of time are recognized:

System–component; kind-of-quantity ($t_1$;$t_2$) = numerical value · unit.

Examples: Patient urine–ammonium; substance rate (8:00;16:00) = 180 nmol/s.

That result may also be reported as an amount-of-substance in the urine sample:

Urine–ammonium; amount-of-substance (8:00;16:00) = 21 μmol.

Patient–vanylmandelate/creatinine; substance rate ratio (6:00;18:00) = 0·06.

That result may also be reported as a substance ratio:

Urine–vanylmandelate/creatinine; substance ratio (6:00;18:00) = 0·06.

*4.4.* A general format is proposed for the presentation of time-related results in clinical chemistry.

*4.4.1.* The format, following previous recommendations, includes the name of the system, the component, the kind-of-quantity, the numerical value, and the unit.

*4.4.2.* When time occurs in the numerator of a 'time' quantity, the word 'time' should be part of the kind-of-quantity name.

Example: Plasma–coagulation, tissue factor induced; time (procedure) = 33 s.

*4.4.3.* When time occurs in the denominator of a derived kind-of-quantity, the word rate should be a part of of the kind-of-quantity name.

Example: Urine–glucose; substance rate = 1 μmol/s.

*4.4.4.* If necessary, for an explicit name of the quantity, the type of process may be given as a specification to the component:

System–component process; kind-of-quantity = numerical value · unit.

Example: Pancreas–amylase production; catalytic rate (30 to 150 min after meal, procedure) = 18 μkat/s (= 18 μmol/$s^2$).

Note that this kind of presentation may be useful especially for metabolic studies of pathophysiological systems in patients, biopsies or isolated cells. It is recommended that the name of the process be placed in parentheses after the component.

### Acknowledgements

Membership of the Commission for varying periods during this report was prepared (1985–1991) was as follows: *Chairman:* H. P. Lehmann (USA); H. Olesen (Denmark); *Titular members:* D. R. Bangham (UK); L. F. Bertello (Argentina); G. Férard (France); J. G. Hill (Canada); M. Lauritzen (Denmark); P. L. Storring (UK); *Associate members:* C. Onkelinx (Belgium); D. J. Campbell (Canada); O. Siggaard-Andersen (Denmark); C. H. de Verdier (Sweden); B. F. Visser (The Netherlands); R. Zender (Switzerland); *National representatives:* J. Breuer (Germany); G. Sotiropoculo (Greece); A. Ferencs (Hungary); N. Montalbetti (Italy); T. Horio (Japan); O. P. Foss (Norway); P. D. Griffiths (UK); C. A. Burtis (USA).

The following people have provided substantial comments: R. Dybkaer (Denmark); J. C. Rigg (The Netherlands).

### Bibliography

1. International Union of Pure and Applied Chemistry. Commission on Quantities and Units in Clinical Chemistry and International Federation of Clinical Chemistry. Expert panel on Quantities and Units in Clinical Chemistry. Dybkaer, R. Approved recommendations (1978). Quantities and units in clinical chemistry. *Clinica Chimica Acta,* **96** (1979), 157F–183F.
2. Bureau International des Poids et Mesures, *Le système International d'Unités (SI).* 6th French and English Edition (Sèvres, 1991).
3. McGlashan, M. L., Manual of symbols and terminology for physicochemical quantities and units. *Pure and Applied Chemistry,* **21** (1970), 3–44.
4. Rigg, J. C., Visser, B. F. and Lehmann, H. P. Nomenclature of derived quantities (Recommendation, 1991). *Pure and Applied Chemistry,* **63** (1991), 1307–1311.

